# Assessment of Two Nutritional Screening Tools in Hospitalized Children

**DOI:** 10.3390/nu12051221

**Published:** 2020-04-26

**Authors:** David Pérez-Solís, Elene Larrea-Tamayo, Cristina Menéndez-Arias, Cristina Molinos-Norniella, Sara Bueno-Pardo, Santiago Jiménez-Treviño, Carlos Bousoño-Garcia, Juan J. Díaz-Martín

**Affiliations:** 1 Pediatrics, Hospital Universitario San Agustín, 33401 Avilés, Spain; david@perezsolis.es; 2 Pediatrics, Primary Care Center Ordizia, 20240 Ordizia, Spain; elene.larreatamayo@osakidetza.eus; 3 Pediatrics, Hospital Alvarez-Buylla, 33611 Mieres, Spain; cristina.menendezaar@sespa.es; 4 Pediatrics, Hospital Universitario de Cabueñes, 33394 Gijón, Spain; cristina.molinos@sespa.es (C.M.-N.); sara.bueno@sespa.es (S.B.-P.); 5 Pediatric Gastroenterology and Nutrition, Hospital Universitario Central de Asturias, University of Oviedo, 33011 Oviedo, Spain; santiago.jimenez@sespa.es (S.J.-T.); cabousono@uniovi.es (C.B.-G.)

**Keywords:** malnutrition, nutrition assessment, risk assessment, hospitalization, STAMP, STRONGkids, child

## Abstract

Aim: to evaluate validity and concordance of Screening Tool for the Assessment of Malnutrition in Pediatrics (STAMP) and Screening Tool for Risk On Nutritional status and Growth (STRONGkids) screening tools for assessment of nutritional risk in pediatric inpatients. Methods: Prospective longitudinal observational multicenter study in children aged 1 month or older admitted as inpatients. Weight, height, cause of admission, demographic data, length of stay, and nutritional interventions were recorded. STAMP and STRONGkids were applied within the first 72 h of admission. Anthropometric measurements were recorded again 12–18 months after admission. Results: Eighty-one patients with median age of 4.1 years completed the study. Agreement between tools was moderate (κ = 0.47). STAMP had a greater tendency to classify patients as high risk (12.3% vs. 2.5%). Both tools showed very weak correlation with height for age. All undernourished patients at the beginning and the end of the study were classified as medium or high risk by STAMP and STRONGkids (100% sensitivity), although specificity was below 50% in all cases. There were no differences in length of stay based on nutritional risk with any of the tools. Conclusions: STAMP and STRONGkids demonstrated moderate agreement, with high sensitivity but low specificity for the diagnosis of undernutrition. Further studies are required to analyze cost-effectiveness of these tools and nutritional interventions derived from them.

## 1. Introduction

Malnutrition is a serious health problem affecting the prognosis of hospitalized patients. Studies carried out in developed countries with adult inpatients have found an association between malnutrition and several outcomes such as the length of hospital stay, readmissions, or mortality [1,2]. Pediatric data are scarce, but a correlation with hospital stay [3] and an increase in infectious complications in patients undergoing major surgery [4] has been observed.

Prevalence of malnutrition in hospitalized children varies significantly depending on the population studied, type of hospital and the method used to assess nutritional status. In the European Union (EU) this prevalence ranges between 2.4% and 26% [5] In Spain, the DHOSPE (*Desnutrición HOSpitalaria en el Paciente pEdiatrico*—Pediatric Undernutrition in Hospitals) study, conducted in 32 hospitals throughout the country, observed a prevalence of moderate or severe malnutrition of 7.8% [6].

In the EU, concern over this issue led the European Council in 2003 to the approval of a resolution on food and nutritional care in hospitals (ResAP 2003-3), which established the need for simple and easy to use nutrition screening tools (NST), and the recommendation to assess nutritional risk in all patients [7]. In 2009 the Prague Declaration further insisted on these needs [8].

NST try to identify patients at nutritional risk, including not only those already malnourished, but also those who can develop malnutrition during admission and, in general, those who can improve their prognosis with a nutritional intervention [9]. The European Society of Pediatric Gastroenterology, Hepatology and Nutrition (ESPGHAN) Committee on Nutrition recommended the systematic use of these tools in hospitalized children [10].

In recent years, several NST have been developed for pediatric use, such as Nutritional Risk Score [11], Pediatric Nutritional Risk Score [12], Subjective Global Nutritional Assessment [4], Paediatric Yorkhill Malnutrition Score [13], Screening Tool for the Assessment of Malnutrition in Paediatrics (STAMP) [14], Screening Tool for Risk On Nutritional status and Growth (STRONGkids) [15], and Pediatric Digital Scaled MAlnutrition Risk screening Tool (PeDiSMART) [16].

The usefulness of these tools is conditioned by their predictive validity to detect nutritional risk, the simultaneous validity among them, their reliability, and their ease of use in clinical practice. There is no consensus on which NST is most suitable for use in hospitalized pediatric patients, mainly due to the lack of a gold standard for the diagnosis of malnutrition [17]. For this study we chose STAMP and STRONGkids as they have been developed in Europe and are the most often validated NST [18].

The objective of this study was to analyze the validity of STAMP and STRONGkids to identify nutritional risk in hospitalized children, as well as the degree of concordance between them.

## 2. Materials and Methods

Multicenter observational, prospective study conducted in three teaching hospitals in Northern Spain: one tertiary and two secondary hospitals. For one month (from June 15th to July 15th) all patients with age equal to or greater than one month of age admitted to the pediatric ward were included. Those cases that did not complete a minimum stay of 24 h or in which the NST could not be applied within the first 72 h of admission were excluded. Patients admitted to pediatric intensive care or to the minor ambulatory surgery unit were not included in the study. Upon admission length/height and weight were recorded naked and in decubitus (infants) or in hospital pajamas and in standing position (children) using calibrated scales, infantometer or stadiometer according to standard procedures. Additional data collected included: age, gender, length of stay (LOS), diagnosis on admission and nutritional intervention performed if any.

In the first 72 h after admission, a pediatrician or pediatric resident with training in gastroenterology and pediatric nutrition was responsible for applying both STAMP and STRONGKids NST (see Supplementary Tables S1 and S2). The STAMP tool [14] combines two questions addressed to caregivers—one on the diagnosis of the child and another on current intake—and also a weight and height assessment in order to establish the nutritional risk. The STRONGKids tool [15] includes two questions addressed to caregivers and two others for healthcare professionals, but it does not include any body measurement. In both cases, the patient is finally categorized in low, medium, or high nutritional risk. Patients were contacted later to perform a new clinical and anthropometric evaluation between 12 and 18 months after hospitalization.

*Z* scores for body mass index (BMI) and height for age (HFA) were calculated according the World Health Organization (WHO) growth charts. Acute malnutrition was defined as a *z* score for BMI less than −2 with normal HFA and chronic malnutrition as a score *z* for HFA less than −2.

The study was approved by the Research Ethics Committee (EPA-05-11). Informed consent was requested from the legal representatives of the patients. Children over 12 years were directly informed by the investigators and personal assent was requested.

### Statistical Analysis

Quantitative variables were expressed by mean and standard deviation or median and interquartile range depending on their normality. Kolmogorov-Smirnov test was used to assess normality of the variables. Qualitative variables were expressed as percentages. Chi-square test was used to assess differences in the distribution of risk groups based on the two NST analyzed. Kruskal–Wallis test was used to analyze differences in the length of stay between the different risk categories. Concordance between both NST was assessed by Cohen’s kappa test. A *p* value < 0.05 was deemed statistically significant. We calculated a sample size of 63 with Epidat 4.2 software (Consellería de Sanidade, Xunta de Galicia, Spain; PanAmerican Health Organization, Washington, DC, USA), assuming a Cohen’s kappa of 0.4, accepting an alpha risk of 0.05 and a beta risk of 0.2 in a two-sided test, with a drop-out rate of 25% during follow-up.

The Statistical Package for the Social Sciences (SPSS Statistics) software version 20 (IBM Corp., Armonk, NY, USA) was used for analysis. Sensitivity, specificity, predictive values, Youden’s J index of diagnostic safety, and odds ratio (OR) for the diagnosis of malnutrition were calculated using a spreadsheet provided by the Spanish Critical Reading Skills Program (CASPe) available at: http://www.redcaspe.org/herramientas/calculadoras.

## 3. Results

Ninety-nine patients were initially included, 81 of whom completed the follow-up (35 girls and 46 boys) with a median age of 4.1 years (range 1 month–16.0 years). Main characteristics of patients included are shown in Table 1. Median hospital stay was 3 days (range 1–24 days). The cause for admission was surgical in 22 cases, 27.2%, (7 appendicitis, 7 thraumatology, 7 otorhinolaryngology, 4 others), infectious in 33 cases, 40.7%, (13 pneumonia, 5 other acute respiratory infections, 5 fever without a focus, 3 urinary tract infections, 3 acute gastroenteritis, and 4 other infections), and other causes in 26 cases (32.1%). At admission, 4 patients (4.9%) had acute malnutrition and 5 (6.2%) chronic malnutrition. Three patients received invasive nutritional support: two through enteral nutrition and one with parenteral nutrition. At the end of the study, 1 patient (1.2%) had acute malnutrition and 5 (6.2%) chronic malnutrition.

**Figure 1:**
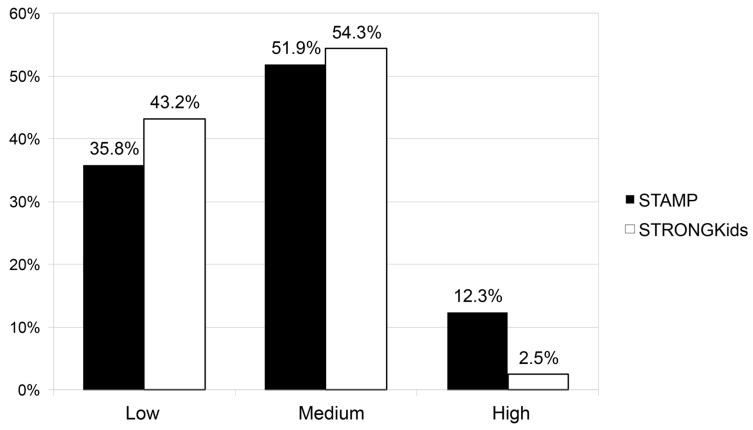
Comparison of the distribution of nutritional risk according to the scale used. STAMP: Screening Tool for the Assessment of Malnutrition in Paediatrics; STRONGkids: Screening Tool for Risk On Nutritional status and Growth.

Stratification of nutritional risk according to the used scale is depicted in Figure 1. Most patients (70.4%) received the same risk classification with both NST. Statistically significant differences between both scales were observed, with a greater tendency for STAMP to classify patients as high risk. Of the 10 patients classified as high-risk by STAMP, only 2 received the same classification by STRONGKids, with the remaining 8 patients classified as medium-risk. Concordance between both NSTs was moderate (Cohen’s kappa = 0.471).

To assess the utility of both scales, their correlation with nutritional status (BMI and HFA) at admission and after 12–18 months was studied. A weak significant correlation was observed only for both tools and HFA (Table 2). Both STAMP and STRONGKids were able to detect all cases of undernutrition at the beginning and end of the study. Both NST classified these patients as high or medium risk (sensitivity 100%, Table 3). However, their specificity to diagnose undernutrition were less than 50% in all possible scenarios (Table 3).

Regarding LOS, no statistically significant differences were observed between patients classified in the different risk levels for both NST (Table 4).

## 4. Discussion

Acute malnutrition prevalence at hospital admission in our study (4.9%) was somewhat lower than that found in the multicenter DHOSPE study conducted in Spain (7.8% for moderate to severe acute malnutrition), although this study used Waterlow index for weight based on national growth tables as indicator [19]. Another multicenter study recently carried out in our country that used BMI as an indicator, using national growth tables instead of WHO growth charts, found a somewhat lower prevalence of acute malnutrition of 3.6% [20]. Regarding chronic malnutrition, the prevalence detected in our study (6.2%) was also higher than the observed in DHOSPE study (4.1%), which used the Waterlow index for height as an indicator. In all these studies, patients were recruited from tertiary and secondary hospitals.

**Figure 2:**
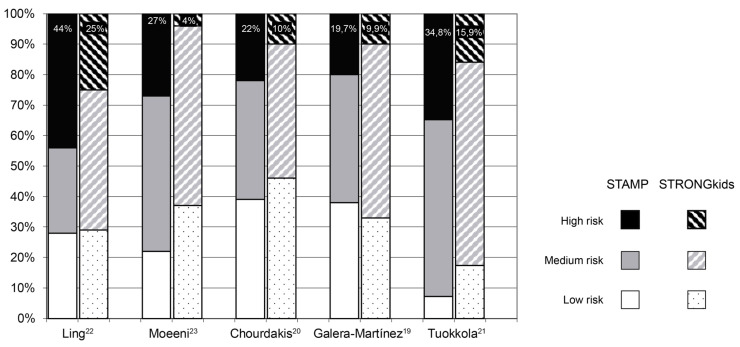
Distribution of nutritional risk in STAMP and STRONGkids in different published comparative studies. Percentages corresponding to patients classified as high risk are shown in figures.

Studies published in recent years comparing STAMP and STRONGKids performance show a low concordance between their results. Chourdakis et al. [21] and Tuokkola et al. [22] observed even lower Cohen’s kappa values than the obtained in our study (0.37 and 0.309 respectively). All published studies consistently show that STAMP classifies many more patients as high risk compared to STRONGKids (Figure 2): 22% vs. 10% in the study by Chourdakis et al. [21], 34.8% vs. 15.9% in Tuokkola et al. [22], 44% vs. 27% in Ling et al. [23], 27% vs. 4% in Moeeni et al. [24], 19.7% vs. 9.9% according to expert observers in Galera-Martinez et al. [20] and 12.3% vs. 2.5% in the present study.

There is currently no consensus on the best method to assess nutritional status in hospitalized children and, therefore, on which gold standard should to be used to validate any NST [18,25]. In our study we used respectively BMI and HFA *Z*-scores as indicators of acute and chronic malnutrition. Considering the categories of moderate and high risk, both STAMP and STRONGKids detected all cases of malnutrition at hospital admission, although with a very low specificity, below 50%. This specificity was lower than the value observed in a recent Finnish study by Tuokkola et al. (69% for STAMP and 89% for STRONGKids, both with 100% sensitivity) [22], although quite similar to those referred in the systematic review of Huysentruyt et al. [25], from studies using other indicators of malnutrition such as weight loss during admission, referral to a nutrition clinic or starting a nutritional intervention.

Another indicator related to nutritional risk is LOS. Several studies have found a greater LOS in hospitalized children with higher nutritional risk [20,21,22,24]. No differences in hospital LOS according to level of nutritional risk were found in our study. Some authors have considered that LOS is not a good indicator for this kind of risk as it might be influenced by many other factors and a causal relationship between both variables has not been demonstrated [25].

As a differential feature in our study, compared to the rest of publications, a prospective follow-up was also carried out with a second evaluation 12–18 months after hospital discharge. At the follow-up visit, a decrease in cases of acute malnutrition was found (from 4.9% to 1.2%), probably reflecting a direct relationship between the nutritional state and the acute process causing hospital admission. However, no change in prevalence chronic malnutrition was observed. The results of STAMP or STRONGKids during admission were not considered in order to indicate nutritional support. Both NST showed the same limitations for predicting malnutrition at follow-up and at hospital admission. It is possible that many patients with acute malnutrition at the time of admission does not benefit from a specific nutritional intervention, as the natural process of recovery after an acute illness may be enough to solve it. More studies are needed to assess this question.

The main limitation of our study is the evaluation of nutritional status based exclusively on basic anthropometric measurements (weight and height), without considering other indicators such as skin folds or body composition. This might have contributed to misclassify some patients as malnourished, especially in the case of chronic malnutrition. Modest sample size, which would limit the power of the study, should also be considered.

There is currently insufficient available evidence to conclude which NST is more accurate in hospitalized children [25,26,27]. Some authors prefer STRONGKids because of its greater simplicity and speed of application, and because of the lower number of patients classified as high risk compared to STAMP [21,23,28]. Although initially designed to be used by different professionals (STAMP for nurses and STRONGKids for physicians), both NST have shown good agreement even when used by any kind of health professionals [20].

Great variability is observed in published studies [18] regarding the method for NST validation. When the objective is to relate nutritional risk to nutritional status at admission, assessments based on somatometry are usually used, such as BMI, weight for height, or HFA. Although these indicators are easy to obtain and are useful for sample description, they have limitations in the individual use as the sole indicator of malnutrition. For this purpose, more complete nutritional assessments that include measures of body composition are more reliable [29]. However, the objective of the NST is not just an approximation to the nutritional status at a given time point, but to establish the risk of developing malnutrition during the process and the need to initiate any nutritional support. This has been attempted to validate through indicators such as the percentage of weight loss during admission, the establishment of nutritional support or referral to hospital dietitians [25], but some of these indicators may respond to different criteria depending their availability among centers. Finally, it would also be necessary to know if nutritional risk at admission is related to a greater probability of malnutrition or complications in the medium term, but, to the best of our knowledge, there are no other published studies that have analyzed the evolution after hospital discharge.

Taking into account the cost of nutritional interventions, it is necessary to know precisely the real usefulness of the NST for the assessment of nutritional status at admission and its relation to the short- and medium-term evolution of hospitalized children. It will also be important to evaluate the efficacy of nutritional interventions derived from their application to improve the prognosis of these patients, which would ultimately be what would justify the widespread of their use.

## 5. Conclusions

STAMP and STRONGKids showed a moderate agreement in classifying nutritional risk in a population of hospitalized children. Both NST detected all cases of malnutrition at admission and after a follow-up of more than one year, although with low specificity. Larger size studies designed to evaluate the cost-effectiveness of the generalized use of these NST and their derived nutritional interventions are needed.

## Figures and Tables

**Table 1:** Characteristics of patients that completed the study.

*n*	81
Age (years)	4.1 (1.3–7.5)
Female (%)	35 (43.2%)
BMI *z*-score	0.24 (−0.80–1.38)
HFA *z*-score	0.21 (−0.57–0.92)
Length of stay (days)	3 (2–5)

BMI: body mass index. HFA: height for age. Data expressed as median (interquartile range) or frequency (percentage).

**Table 2:** Correlations between STAMP and STRONGkids tools and body mass index (BMI) and height for age (HFA) *z* scores.

		Admission		Follow−Up	
NST	Index	Spearman’s Rho	*p*	Spearman’s Rho	*p*
STAMP	BMI	−0.044	0.698	0.022	0.844
STRONGkids	BMI	−0.159	0.156	−0.003	0.978
STAMP	HFA	−0.213	0.056	−0.220	0.049 *
STRONGkids	HFA	−0.290	0.009 *	−0.285	0.010 *

NST: Nutrition Screening tool. STAMP: Screening Tool for the Assessment of Malnutrition in Paediatrics; STRONGkids: Screening Tool for Risk On Nutritional status and Growth. * *p* < 0.05.

**Table 3:** Diagnostic efficacy parameters for both tools to detect acute and/or chronic malnutrition considering those classified as medium or high risk. In brackets, the confidence intervals for 95% are expressed.

	Admission		Follow-Up	
	STAMP	STRONGKids	STAMP	STRONGKids
Sensitivity	100% (70.1–100%)	100% (70.1–100%)	100% (61.0–100%)	100% (61.0–100%)
Specificity	40.3% (29.7–51.8%)	48.6% (37.4–59.9%)	38.7% (28.5–50.0%)	46.7% (35.8–57.8%)
PPV	17.3% (9.4–29.7%)	19.6% (10.7–33.2%)	11.5% (5.4–23.0%)	13.0% (6.1–25.7%)
NPV	100% (88.3–100%)	100% (90.1–100%)	100% (88.3–100%)	100% (90.1–100%)
Youden’s J Index	0.40	0.50	0.40	0.50
PLR	1.67 (1.39–2.02)	1.95 (1.55–2.44)	1.63 (1.36–1.95)	1.88 (1.52–2.32)
NLR	0.0 (0–NC)	0.0 (0–NC)	0.0 (0–NC)	0.0 (0–NC)

NC: not calculable. PPV: positive predictive value. NPV: negative predictive value. PLR: positive likelihood ratio. NLR: negative likelihood ratio.

**Table 4:** Length of stay (median, interquartile range) in different categories of risk for both nutrition screening tools.

NST	Low Risk	Medium Risk	High Risk	*p* *
STAMP	3 (2–4)	4 (2–5)	2.5 (2–3.25)	0.24
STRONGKids	3 (2–4)	4 (2–5)	2 (2–2)	0.23

* Kruskal–Wallis test for independent samples. NST: Nutrition screening tool.

## Supplementary Materials

The following are available online at https://www.mdpi.com/2072-6643/12/5/1221/s1, Table S1: First four steps of Screening Tool for the Assessment of Malnutrition in Paediatrics (STAMP). Original tool also includes a fifth step about developing a care plan based on the child’s overall risk of malnutrition (http://www.stampscreeningtool.org), Table S2: Screening Tool for Risk On Nutritional status and Growth (STRONGkids) [15].
