# Supplementary material for: Assessment of Two Nutritional Screening Tools in Hospitalized Children
Source: Nutrients. 2020 Apr 26;12(5):1221. doi: 10.3390/nu12051221 (PMC7281986; doi:10.3390/nu12051221)
Supplement: Supplementary file 1 [file nutrients-12-01221-s001.pdf]

Supplementary Materials

Table S1. First four steps of Screening Tool for the Assessment of Malnutrition in Paediatrics (STAMP). Original tool also includes a fifth step about developing a care plan based on the child's overall risk of malnutrition (http://www.stampscreeningtool.org).

**Step 1 – Diagnosis**	
**Does the child have a diagnosis that has any nutritional implications?**	Score
Definitely	3
Possibly	2
No	0
**Step 2 – Nutritional intake**	
What is the child's nutritional intake?	Score
None	3
Recently decreased/poor	2
No change/good	0
**Step 3 – Weight and height**	
Use a growth chart or the centile quick reference tables to determine the child's measurements	Score
> 3 centile spaces/ ≥ 3 columns apart (or weight < 2nd centile)	3
> 2 centile spaces/ ≥ 2 columns apart	1
0 to 1 centile spaces/columns apart	0
**Step 4 – Overall risk of malnutrition**	
Add the scores from steps 1–3 together to calculate the overall risk of malnutrition	Score
High risk	≥4
Medium risk	2-3
Low risk	0-1

Table S2. Screening Tool for Risk On Nutritional status and Growth (STRONGkids) [15].

**1. Subjective clinical assessment**	
**Is the patient in a poor nutritional status judged by subjective clinical assessment (diminished subcutaneous fat and/or muscle mass and/or hollow face)?**	Score
Yes	1
No	0
**2. High risk disease**	
Is there an underlying illness with a risk of malnutrition or expected major surgery?*	Score
Yes	2
No	0
**3. Nutritional intake and losses**	
Are one of the following items present?<br>• Excessive diarrhoea (≥5 per day) and/or vomiting (>3 times/day) the last few	Score

days?<br>• Reduced food intake during the last few days before admission (not including fasting for an elective procedure or surgery)?<br>• Pre-existing dietetically advised nutritional intervention?<br>• Inability to consume adequate intake because of pain?	
Yes	1
No	0
**4. Weight loss or poor weight gain?**	
Is there weight loss or no weight gain (infants <1 year) during the last few weeks/months?	Score
Yes	1
No	0
**Total score**	
Calculate total score (total of items 1-4)	Score
High risk	4-5
Medium risk	1-3
Low risk	0

The first 2 items were assessed by a pediatrician and the second 2 items were discussed with the parents or caregivers. Questions answered with 'unclear' were classified as 'no'.

* High risk diseases:

- Anorexia nervosa
- Burns
- Bronchopulmonary dysplasia (maximum age 2 years)
- Celiac disease
- Cystic fibrosis
- Dysmaturity/prematurity (corrected age 6 months)
- Cardiac disease, chronic
- Infectious disease (AIDS)
- Inflammatory bowel disease
- Cancer
- Liver disease, chronic
- Kidney disease, chronic
- Pancreatitis
- Short bowel syndrome
- Muscle disease
- Metabolic disease

- Trauma
- Mental handicap/retardation
- Expected major surgery
- Not specified (classified by doctor)
